# Commentary: Automation of laboratory instrumentation: where is it going?

**DOI:** 10.1155/S146392468000020X

**Published:** 1980


### Automation of laboratory instrumentation: where is it going?

The editors of *The Journal of Automatic Chemistry,* as individuals, have been exercising their thoughts for some years about where automation is leading. It was in this context that they discovered that they were not alone but rather that there were a number of experts in the field who cared about the goals and approaches of automation. From this arose the idea of bringing forth *The Journal of Automatic Chemistry,* in spite of the fact that there were many voices crying "what do we need another journal for?"

In the editorial columns we have often discussed what automation in analytical chemistry is about and what its large scale effects are. Automation is a systems problem and therefore has wide ranging consequences. Undoubtedly we have to concern ourselves with the immediate effects of automation. The social implications may well be such that automation has to be brought, albeit temporarily, to a halt. In the longer run, however, none of us really believes that anything will be able to prevent further development.

It is in this context that we have to ask ourselves in which direction development will go. Although it is always hard to make long-term predictions, there are usually strong indications available about what is going to befall us. As far as automation in analytical chemistry is concerned the signs are written on the wall, and the editors of *The Journal of Automatic Chemistry* would like to feel that they represent part of that wall. The papers that are published should reflect what the future is holding in store for us.

We would like to be more definite about this topic. After all, we are living in the age of the electronic revolution and the microprocessor. We would like to offer our opinions on what the proliferation of increasing computer power and falling prices will make available to us.

First of all, and this may disappoint some people, it will bring more of the same. There will be greater ease of operation, more push-button functions, more automatic programs for evaluation, more processes that may be controlled. This will continue for some time, and prices will continue to fall.

Although this development may be important for our immediate future, it will not be of fundamental significance. Significant developments come about only in situations where 1+1 equals 3. This means where, out of a multitude of possibilities, some new feature arises that so far has been unforseeable.

Now it is, indeed, very hard to predict the future on this basis. Naming it is inventing it. But it certainly should be possible to give an indication of the direction in which we are going. The increased importance of software and memory capacity – it is estimated that in new instrumental developments for analytical chemistry these will make up as much as 50% of the development cost – will allow us to dwell on the field of cybernetic engineering. That is to say, we will approach instrumentation from a behavioural point of view.

As a first step, instruments in analytical chemistry will be equipped with a "training" or "learning" feature. Most analytical procedures are the result of an optimisation procedure. A machine can do this automatically and rapidly if we know how to implement it. Although the machine does not show behaviour in the strict sense of the word, we may interpret it as such if it produces results that are contrary to "what we might expect intuitively". For the designer the task is still a comparatively simple one : program and implement a known optimisation technique.

Real learning, however, assumes the evaluation of past experiences. Instead of proceeding on a predetermined basis an empirical basis is chosen. Past successes and failures are recorded and retained for future use. Although we are still operating in a deterministic realm (the same past together with the same current conditions will produce the same outcome) it becomes very hard to predict behaviour. The designer faces a much harder problem. He has to program the learning technique and will not be able to predict behaviour in an individual situation. Such machines will perform wonderfully in a more or less constant environment but in extreme or abnormal situations erratic behaviour may occur. The dangers that may ensue from this have long been recognised. We may no longer be able to discern in sufficient time whether a decision by a machine is going to be superclever or super-stupid.

As long as we stay in the field of analytical chemistry and are able to cross-check results we stand a fair chance of containing these dangers. It is predictable, therefore, that learning techniques will spread quickly to analytical chemistry. Hardware will evolve into multiprocessor systems but the software task looks insurmountable at present. Adaptive control theory will have to be developed further and become essential knowledge for programmers and engineers.

It's a long way to go. But, like evolution, it is unavoidable. We had better prepare now to live with it. This should make the difference between a bright and a gloomy future.

The difference from true evolutionary behaviour is, of course, that we are still operating in a deterministic manner. Only if we admit random elements to broaden purposely the basis of experience can we get closer to evolution.

This is even more far fetched. Extrapolation of the rate of development indicates that we may be there sooner than we anticipated. Parallels can be drawn with genetic engineering where living systems are manipulated. Some dangers have been recognised and precautionary steps have been taken, nevertheless development is going ahead at full speed. There the future has started. In automation it will come too.

*R.W. Arndt*

### From the Editor's desk

### International coverage of the Journal

In many respects *The Journal of Automatic Chemistry* achieves the objectives laid down some two years ago when it first emerged from an embryonic state into a fully equipped publication. From the outset one of the main aims has been to have an international base. The editorial board and the corresponding editors cover many of the countries throughout the globe as well as the full range of disciplines covered in the term automatic analytical chemistry. We will continue to update the editorial coverage to widen the scope and international nature of the board. This month we are pleased to wellcome Dr Joseph X. Dautlick to the team knowing that it will be strengthened by his experience. The technical articles published have reflected a very international aspect. This issue for example includes the first paper from France which has long been overdue. Automation is a world wide problem although there are undoubtedly many national nuances.

In one respect we have so far failed to achieve an international flavour and that is in the coverage of meetings and in editorial relating to new equipment. In the first respect hopefully we can encourage corresponding editors to prompt

